# Comparison of Physicochemical Properties of Silver and Gold Nanocomposites Based on Potato Starch in Distilled and Cold Plasma-Treated Water

**DOI:** 10.3390/ijms24032200

**Published:** 2023-01-22

**Authors:** Magdalena Janik, Karen Khachatryan, Gohar Khachatryan, Magdalena Krystyjan, Zdzisław Oszczęda

**Affiliations:** 1 Faculty of Food Technology, University of Agriculture, ul. Balicka 122, 30-149 Kraków, Poland; 2 Nantes Nanotechnological Systems, Dolnych Młynów Street 24, 59-700 Bolesławiec, Poland

**Keywords:** starch, nanometals, nanocomposite, plasma water

## Abstract

Nanometal-containing biocomposites find wide use in many industries and fields of science. The physicochemical properties of these materials depend on the character of the polymer, the size and shape of the metallic nanoparticles, and the interactions between the biopolymer and the nanoparticles. The aim of the work was to synthesise and study the effect of plasma-treated water on the properties of the obtained metallic nanoparticles as well as the physicochemical and functional properties of nanocomposites based on potato starch. The metallic nanoparticles were synthesised within a starch paste made in distilled water and in distilled water exposed to low-temperature, low-pressure plasma. The materials produced were characterised in terms of their physicochemical properties. Studies have shown that gold and silver nanoparticles were successfully obtained in a matrix of potato starch in distilled water and plasma water. SEM (Scanning Electron Microscopy) images and UV-Vis spectra confirmed the presence of nanosilver and nanosilver in the obtained composites. On the basis of microscopic images, the size of nanoparticles was estimated in the range from 5 to 20 nm for nanoAg and from 15 to 40 nm for nanoAu. The analysis of FTIR-ATR spectra showed that the type of water used and the synthesis of gold and silver nanoparticles did not lead to changes in the chemical structure of potato starch. DLS analysis showed that the nanoAg obtained in the plasma water-based starch matrix were smaller than the Ag particles obtained using distilled water. Colour analysis showed that the nanocomposites without nanometals were colourless, while those containing nanoAg were yellow, while those with nanoAu were dark purple. This work shows the possibility of using plasma water in the synthesis of nanometals using potato starch, which is a very promising polysaccharide in terms of many potential applications.

## 1. Introduction

Nanotechnology is among the fields of science that have enjoyed the most dynamic growth in the last few decades [1,2,3,4]. Scientists have observed that nanoscale materials have unique properties, unlike their macroscale equivalents. Nanoscale materials can exhibit different physicochemical properties, such as colour, solubility, strength or toxicity, stemming primarily from their nanometric size [5,6]. The dynamic development of nanotechnology has been amplified by the emergence of instruments that enable both observing and manipulating nanometre-sized structures [7] and with the development of modern research methods [7,8]. The main area where nanotechnology is used in the food industry is developing ingredients that provide targeted assimilation or new functional properties for food. Furthermore, nanotechnology has found wide use in developing nanosensors for monitoring food freshness [9,10]. Using modern modification methods, functional food can be created, for example, using nanocapsules containing active substance delivery systems [11,12,13]. In addition, there are metal nanoparticles on the market that are sold as dietary supplements [14,15]. Nanosensors are mainly used in developing innovative food packages, which are termed active and smart packaging systems. Their purpose is to extend shelf life, minimise food spoilage and maintain the required quality and safety standards at all stages of the food marketing chain [16,17,18,19,20].

Many scientific studies focus on developing composite materials—combinations of polysaccharides and nanoparticles—due to their improved physicochemical, biological and mechanical properties, among others [21]. In the food industry, nanosilver has been applied in developing food packages and containers to take advantage of its anti-microbial properties. The mechanism of anti-mycobiological action of nanosilver consists in damaging the cell wall of microorganisms, which results in increased membrane permeability. As a consequence, there is an increase in permeability, and thus a disturbance in the economy of the bacterial cell, and ultimately leads to damage to its genetic material [22,23,24]. Currently, there is a large number of nanosilver-based antibacterial packaging materials that can be found on the global market [25,26,27,28]. Additionally, many studies have demonstrated that nanoparticle-containing polymer materials exhibit good biocidal properties [28,29]. From this perspective, developing materials containing this type of nanostructures appear to be a highly promising technological solution [30]. Another, somewhat less popular, element used to make nanomaterials is gold. Gold nanoparticles, due to their properties, have great potential for biomedical, environmental and industrial applications. According to literature data, AuNPs are biocompatible; they can bind to proteins, affecting their biological activity [31,32]. Gold nanoparticles are inert and have a high resistance to surface oxidation [33]. Moreover, gold nanoparticles show an antibacterial, antifungal and anticancer effect [33,34,35]. Potato starch is one of the most common polysaccharides of plant origin found in nature [36]. Due to its properties, such as natural sourcing, renewability, low price and non-toxicity, biodegradability, gelling and thickening characteristics, it has been widely used in the food industry for many years and continues to be the object of numerous studies [37]. It has also been demonstrated that potato starch forms a good matrix for producing metal nanoparticles, stabilises the nanometals produced in it, and prevents their aggregation [19,20,32,38,39,40,41].

Water is the main, most familiar and most widespread chemical compound on our planet. Due to its unique properties, it constitutes a common source of scientific research [42]. Water can be subjected to various modifications that change its structure forming dimers, clusters or gigaclusters, affecting its properties [43]. When exposed to a low-temperature, low-pressure, low-frequency glow plasma, water undergoes changes in its structure and physicochemical properties, thus providing an interesting base for developing innovative solutions in many fields [44,45]. Kravchenko et al. [46] showed that under the influence of low-temperature low-pressure plasma, water and saline significantly changed their properties, such as pH, electrical conductivity and surface tension. Studies have been published on the use of plasma-treated water in brewing [47,48], in bacteria control and for improved sterilisation efficiency [49], and in various areas of agriculture [50,51,52]. It has also been demonstrated that cold plasma increases the surface energy and hydrophilicity of starch granules [53].

Under this project, we prepared nanocomposite films containing nanogold (Au) and nanosilver (Ag) in a potato starch-based (PS) polysaccharide matrix produced using distilled water and plasma-treated water. We characterised the resulting nanocomposites in terms of their physicochemical properties. The morphology and size of the resulting nanoparticles were determined with scanning electron microscopy (SEM) and dynamic light scattering (DLS). The properties of the composites were characterised using FTIR-ATR (Fourier transform infrared spectroscopy—attenuated total reflectance) and UV-Vis (Ultraviolet-visible spectrophotometry). Spherical nanoparticles of silver and gold were produced, and the application of plasma water affected the polydispersity, colour and transparency of the resulting composites.

## 2. Results and Discussion

### 2.1. UV-Vis

**Figure 1:**
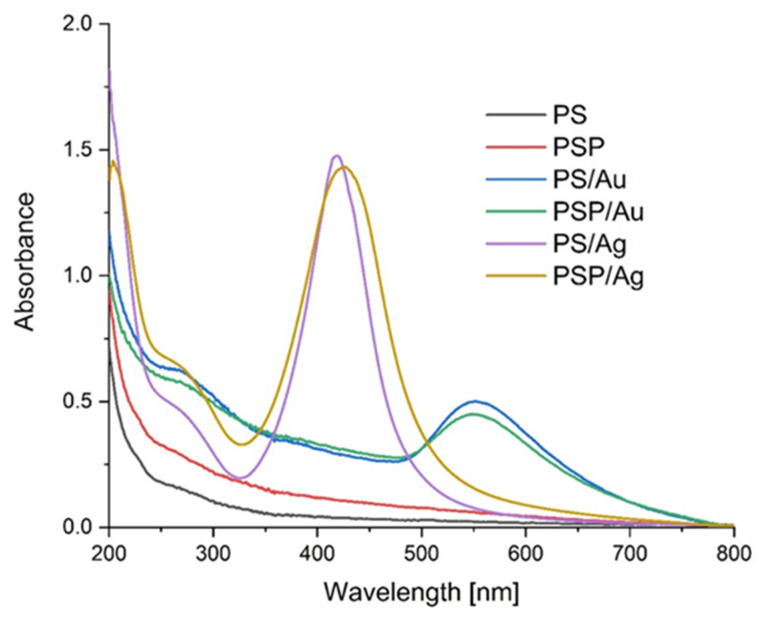
UV-Vis spectra of the obtained films.

Potato starch paste in distilled water (PS) and in plasma water (PSP) was used as the matrix for preparing nanoAg and nanoAu composites. When AgNO_3_ and HAuCl_4_ were added to the PS and PSP suspensions, the reaction mixtures turned yellow and dark purple, respectively, both in distilled and in plasma water, which suggested that nanoparticles of silver and gold, respectively, had formed. The UV-Vis spectra recorded for the PS, PSP and PS/Ag, PSP/Ag and PS/Au, PSP/Au composites are shown in Figure 1. The spectra recorded for the nanoAg and nanoAu composites show a characteristic absorption maximum of Ag and Au nanoparticles, centred at 430 nm and 550 nm, respectively. The centre positions and bandwidths indicate that the nanoparticles formed are of different sizes. Differences stemming from the type of water used were observed. Differences can be observed in the structure and position of the absorption bands of the nanoAg particles generated in distilled and plasma water. The resulting UV-Vis spectra suggest that nanometals produced within the same polysaccharide matrix but using different water types differ from each other. These changes may be a consequence of the effects of the plasma water on the starch structure [54,55]. For silver, the nanoparticles generated are larger in the plasma water-based matrix. In the case of gold, on the other hand, no notable changes in nanoparticle size were observed. Many literature sources describe relationships between nanoparticle size and their UV-Vis spectra [56].

### 2.2. FTIR-ATR Spectrophotometry of the Composites Obtained

**Figure 2:**
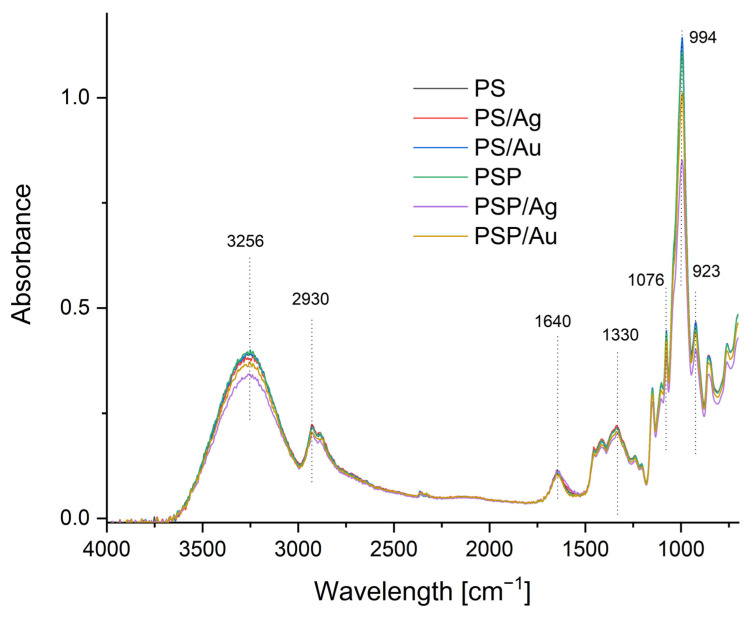
FTIR-ATR spectra of the obtained nanocomposites.

The FTIR-ATR spectra enabled the chemical structure of the nanocomposites produced to be analysed. The obtained spectra are shown in Figure 2. Bands characteristic of potato starch can be identified in the spectra. The first strong and broad absorption band at 3256 cm^−1^ results from the presence of –OH groups in the potato starch structure. The H–O–H absorption peak at 1635 cm^−1^ comes from tightly bound water present in the starch, and the peak at 2936 cm^−1^ is identified with C–H stretching vibrations. The peaks at 923, 994 and 1076 cm^−1^ represent C–O–C stretching vibrations in the starch structure [57].

### 2.3. Scanning Electron Microscopy (SEM)

**Figure 3:**
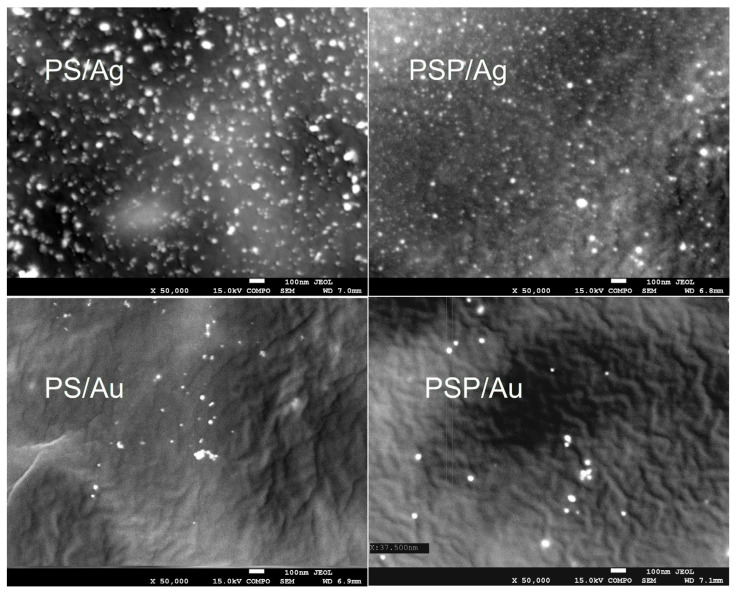
SEM of the obtained nanocomposites.

**Figure 4:**
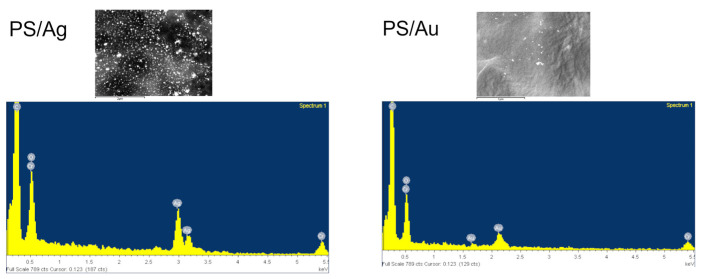
EDS spectra for PS/Ag and PS/Au nanocomposites.

Figure 3 shows SEM images of the obtained composites. The test confirmed that we obtained spherical nanoparticles sized from 5 to 20 nm for Ag and 15 to 40 nm nanoparticles for Au. Figure 4 shows an EDS analysis for selected samples (PS/Ag and PS/Au), which confirms the presence of gold and silver in the nanocomposites produced.

### 2.4. Particle Size of Silver and Gold Particles

The size of the particles and aggregates was measured for aqueous nanocomposite solutions by placing 1 mL gel in a cuvette. Aggregates sized 536–924 nm and likely sedimenting particles with sizes exceeding 1125 nm, up to 1431 nm, were observed in the samples (Table 1). A DLS analysis showed that the nanoAg particles obtained in the plasma-water based starch matrix were smaller than the Ag particles obtained using distilled water. An opposite relationship is visible for nanoAu; the gold particles in the plasma water-based matrix were substantially larger. It can be assumed that the water type affects the polysaccharide structure, which results in an ordering of the nanoparticles in the polysaccharide matrix. Two peaks were observed in the 10–100 nm area and above 100 nm in the DLS spectra for the PS/Ag sample. For PSP/Ag, 4 peaks were present in the 10–10,000 nm section. For the PS/Au nanocomposite, 3 peaks were obtained within the 10–100 nm range, and a peak indicating the presence of larger aggregates was found above 100 nm. The PSP/Au nanocomposite spectrum showed four peaks between 10 and 10,000 nm. It can therefore be surmised that nanometals obtained in a plasma water-based composite have varied sizes. The DLS spectrum results generated correlated with the UV spectrum measurement results. It must be remembered that the DLS result provides the size of the nanoparticle and polysaccharide combination structures. Furthermore, producing gold nanoparticles requires the use of a strong acid, which can cause small changes in the polysaccharide structure, creating more free spaces where aggregation can occur. The DLS spectra (Figures S1–S4) are provided in the Supplementary Data section.

### 2.5. Water Properties

The water properties of the obtained nanocomposites are presented in Table 2. The water content (WC) in the control samples was 13.02% and 15.09% for PS and PSP, respectively. For the batch containing nanoAg, the water content was 12.17% for the PSAg group and 13.26% for PSP/Ag. The batch containing nanoAu was characterised by the water content of 11.46% and 14.12% for PS/Au and PSP/Au, respectively. On the other hand, for water solubility (WS), the values for all groups ranged from 26.22% to 28.34%, depending on the group. For films containing Ag NPs, the water solubility was 26.22% for PS/Ag and 27.66% for PSP/Ag. The films with a nanoAu addition had a solubility within the 28.34% and 26.55% range. Similar results were obtained by Hu et al. [37], who studied films made with oxidised potato starch. They obtained similar water solubility values for the films, within the range of 22.77% to 29.86%. The results obtained indicate that the presence of the nanometals did not affect the film water solubility.

CO_2_ permeability increases slightly in composites that contain nanometals, but there is no statistically significant difference depending on water type. Water permeability, on the other hand, is almost identical for all groups.

### 2.6. Mechanical Properties

The thickness of the samples varied from 0.094 mm to 0.124 mm (Table 3). The addition of Ag and Au to the PS composites increased their thickness by approx. 12 and 17%, respectively, and 20–31% in nanocomposites with plasma-water samples. According to Krystyjan et al. [58], it may have resulted from the solid content enrichment in the samples. The use of plasma water (PSP sample) reduced the starch film thickness by approx. 11%. There are no statistically significant differences between the samples containing metal nanoparticles. When the mechanical parameter values were compared, a statistically significant effect of the Ag and Au on the starch film strength (TS) was observed. For plasma water-based starch films, the presence of metals weakened the breaking strength of the films. It must be stressed, though, that the observed changes were minor. On the other hand, when plasma water was used, nanoAg improved film strength, while nanoAu degraded this parameter. A similar relationship was noted for the other mechanical parameter, tensile strength. Composite breaking and tensile strength are important parameters that are used to assess a composite’s ability to maintain integrity in the presence of varied environmental factors. Therefore, these parameters indicate a specific possible application for the nanomaterials in packages and other industries. High tensile strength is valued especially in applications where a material needs to provide structural integrity or strengthen the structure of the packaged products, so changes in shape and susceptibility to deformations are undesirable [58,59].

### 2.7. Colour, Opacity and Appearance Properties of the Prepared Nanocomposites

The colour and opacity parameters of the composites are shown in Table 4. The opacity results show that the control films were characterised by high transparency and clarity compared to the films containing nanometals. PS and PSP/Ag show similar values. Differences in the control batch and PS/Ag are also visible. The higher O value for the non-plasma water-based composites indicates a lower transparency level of the films. For films with Ag NPs, the opacity results were 7.02 and 5.49 for PS/Ag and PSP/Ag, respectively. For films containing nanoAu, the opacity values were 8.14 in distilled water and 10.39 in plasma water. These values indicate that the presence of nanometals, in particular nanogold, may prevent penetration by UV light, so such films may find applications as an excellent barrier against UV radiation. According to literature data, the yellow colour of the composites that contained silver nanoparticles shows that the nanoparticles produced were stable [60]. The values of the L* parameter were the highest for both control samples (PS and PSP); this parameter measured the brightness of the composites. The incorporation of Ag and Au nanoparticles into the structure of the composites resulted in decreased brightness. The incorporation of Ag nanoparticles into the starch matrix decreased this parameter by about 25% for composites based on regular water and by 30% for composites containing plasma water. The effects of the Au nanoparticles were even more pronounced, as composite colour darkening by 51% and 53%, respectively, was observed. All the Ag nanocomposites had a higher share of red (a* > 0) and yellow (b* > 0) than the control composites. The addition of Au nanoparticles increased the a* parameter and decreased the b* values, indicating a tendency towards a red and blue colour. The effects of using plasma water on the colour parameters were visible only for composites with metal nanoparticles. When comparing the obtained results with the literature data, it should be noted that the obtained composites with Ag and Au nanoparticles were more transparent than the commonly used LDPE (low-density polyethylene) and PVDC (polyvinyl dichloride) synthetic films [61]. The dominant effect of the nanoparticles on the composite colour results from the size and shape of the nanoparticles produced, as these parameters determine how the nanoparticles interact with light [62].

**Figure 5:**
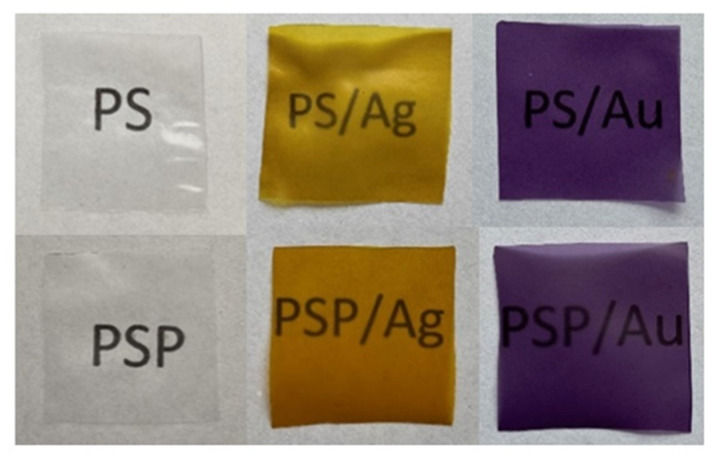
Appearance of the obtained nanocomposites.

The appearance of the films produced is shown in Figure 5. Based on the visual assessment, it can be said that the starch composites were colourless and characterised by complete transparency. NanoAg-containing nanocomposites, on the other hand, were yellow, while those with nanoAu were dark purple.

## 3. Materials and Methods

### 3.1. Materials

Potato starch (Sp) (amylose: amylopectin ratio = 26: 74; 12% moisture and 550 ppm phosphorus) was purchased from Pepees SA, Łomża, Poland. Glycerine (Sigma-Aldrich, 99.5%); AgNO_3_ (Sigma-Aldrich, Poznań, Poland, 99.99%); HAuCl_4_·H_2_O (Sigma-Aldrich, 99.9%). NaOH (99.5% p.a) and HCl (37% p.a.) were purchased from Chemland (Chemland, Stargard, Poland). None of the chemical reagents had been subjected to prior purification before being used in the experiments.

### 3.2. Methods

#### 3.2.1. Preparation of Plasma Treated Water

Plasma water was prepared according to the method described in earlier works [44,63]. Water (2000 mL) in a glass volumetric flask was placed in the chamber of the reactor [63,64] and exposed to Glow Plasma for 30 min. Plasma of 38 °C was generated at 5 × 10^−3^ mbar, 600 V, 50 mA and 280 GHz frequency. Conductivity, pH and oxygen content were, respectively: 2.32 µS, 5.56 and 61.93% (per cent of saturation) for distilled water and 1.46 µS, 5.19 and 63.53% for plasma-treated distilled water.

#### 3.2.2. Preparation of Nanocomposites

Potato starch (15.0 g) was suspended in distilled water (485.0 mL), PS batch. The same procedure was applied for the plasma water (PSP). The resulting suspensions were heated in a water bath placed on a magnetic stirrer (Heidolph MR3002, Schwabach, Germany) at 65–70 °C for 2 h to produce a starch paste. Silver (PSAg) and gold (PSAu) nanocrystals were generated by adding either 26.0 mL AgNO_3_ (0.01 mol/L) or 13.8 mL HAuCl_4_ (0.01 mol/L) solution and 10.0 mL 4% D-xylose solution as a reducer to the starch paste. The solutions were constantly stirred for 30 min, and then 7.5 g glycerine was added as a plasticiser. The reaction mixtures were stirred at the same temperature for 24 h. The resulting solutions were cooled down to 40 °C and poured into Petri dishes (120 × 120 mm), and then left to dry in the air at room temperature. The dried films were stored in sealed containers.

#### 3.2.3. Water Content and Solubility

Five randomly selected samples of each type of film (2 × 2 cm) were weighed (to the nearest 0.0001 g), and the starting weight (W_1_) was recorded. The samples were then dried in an oven at 130 °C for 2 h to determine the initial dry weight (W_2_). After drying, each film was dipped into 10 mL of distilled water, tightly covered and stored for 24 h at room temperature (25 ± 2 °C). After this time, all samples were dried with filter paper and weighed (W_3_). Five measurements were carried out for each film sample to calculate the mean value. Water content and film solubility were calculated from the following equations:

(1) $$\mathrm{Water}\;\mathrm{content}\;\left(\mathrm{WC}\right)=\frac{W_{1}-W_{2}}{W_{1}}\times 100\%$$

(2) $$\mathrm{Solublity}\;\left(\mathrm{WS}\right)=\frac{W_{2}-W_{3}}{W_{2}}\times 100\%$$

#### 3.2.4. Water Vapour Transmission

To perform the CO_2_ and H_2_O permeation experiment, 36 special flasks with plugs having an 8 mm diameter hole were prepared. Approx. 1 g sodium hydroxide (NaOH) pellets were placed in the above containers. 1 cm diameter samples were cut from the nanocomposites using a cork borer and tightly put on the previously prepared containers so that permeation could occur only on the test film surface. On day zero (D0), the mass of the container + plug, plug, the mass of the NaOH added, and the mass of each film disk (M1, M2, M3) were weighed. The permeation measurements were taken over two weeks at room temperature (25.0 °C ± 2.0 °C) by inspecting the mass increase every 3 days. On the last day of the experiment, the contents of each flask were moved quantitatively to 100 cm^3^ volumetric flasks, thoroughly mixed, and 2 mL of the solution was sampled and titrated with 0.1000 mol/L hydrochloric acid (HCl) in the presence of phenolphthalein and methyl orange. Each test group was assayed in triplicate.

The Na_2_CO_3_ was calculated using the following equation:

$${\mathrm{mNa}}_{2}{\mathrm{CO}}_{3}=\frac{2{\;*\;b\;*\;c}_{\mathrm{HCl}}{\;*\;M}_{{\mathrm{Na}}_{2}{\mathrm{CO}}_{3}}\;*\;W}{2}$$

where:

a—HCl volume used for titration in the presence of phenolphthalein [l]
b—HCl volume used for titration in the presence of phenolphthalein [l]
C_HCl_—concentration of HCl acid [mol/l]
M_Na2CO3_—Na_2_CO_3_ molar mass
W—flask and pipette commensurability

#### 3.2.5. Mechanical Properties of the Composites

The samples for mechanical analyses were prepared according to ISO standards [65] and assayed using a TA-XT plus texture analyser (Stable Micro Systems, Haslemere, UK). The composites were cut into 35 mm × 6 mm strips and placed in holders. The initial grip distance between holders was 20 mm, and the rate of grip separation was 2 mm/min. The tensile strength (TS) was calculated by dividing the tensile force (maximum force at rupture of the film) by the cross-sectional area of the film. The percentage elongation at break (E) was calculated by dividing the elongation at rupture by the initial gauge length and multiplying by 100. The reported results were the average values of ten replicates.

#### 3.2.6. Thickness Measurement

The thickness of the composites was measured with a micrometer, catalogue no. 805.1301 (Sylvac SA, Crissier, Switzerland), with a 0.001 mm resolution. The sample thickness was the average of five measurements performed in various places within the gauge length area.

#### 3.2.7. Surface Colour Measurements

The surface colour was tested with the use of a Konica MINOLTA CM-3500d device (Konica Minolta Inc., Tokyo, Japan) with a 30 mm diameter window, using a reference D65 illuminant/10° observer. The results were expressed using the CIELab system. The following parameters were determined: L* (L* = 0 black, L* = 100 white), a*—share of green (a* < 0) or red (a* > 0), b*—share of blue (b* < 0) or yellow (b* > 0). The measurements were taken against a white background standard. The experiment was repeated five times.

#### 3.2.8. UV-Vis Absorption Spectrophotometry and Opacity

The UV-Vis absorption spectra of the nanocomposites were recorded using a Shimadzu 2101 (Shimadzu, Kyoto, Japan) scanning spectrophotometer in the 200–800 nm range. For the measurements, the films were placed in a 10 mL quartz cuvette, with an empty cuvette used as a reference. The opacity was also determined at a wavelength of 600 nm according to the following equation:

$$\mathrm{Opacity}=\frac{\mathrm{Abs}\;600}{x}$$

where Abs 600 is the absorbance value at 600 nm, and x is the film thickness (mm).

#### 3.2.9. FTIR-ATR Spectrophotometry

The FTIR-ATR spectra of the composites were analysed using a MATTSON 3000 spectrophotometer (Madison, WI, USA) within the 4000–700 cm^−1^ range at a resolution of 4 cm^−1^. The spectrophotometer was equipped with a 30SPEC 30 Degree Reflectance adapter (MIRacle ATR, PIKE Technologies Inc., Madison, WI, USA).

#### 3.2.10. Scanning Electron Microscopy (SEM)

The size and morphology of the gold and silver nanoparticles in the produced composites were analysed using a JEOL 7550 (Akishima, Tokyo, Japan) scanning electron microscope equipped with an EDS analyser for local chemical analysis.

#### 3.2.11. Particle Size Analysis

The particle size of the produced nanoparticles was assessed at 25 °C via dynamic light scattering (DLS) (Zetasizer Ultra Red, Malvern Instruments Ltd., Worcestershire, UK).

#### 3.2.12. Statistical Analysis

The experimental data were subjected to an analysis of variance at the confidence level of *p* = 0.05 using the Statistica v8.0 software (Statsoft, Inc., Tulsa, OK, USA). The Fisher’s test was used to determine the statistically significant differences.

## 4. Conclusions

Spherical nanoparticles of gold and silver were successfully produced in a potato starch matrix in distilled and plasma water. Based on microscope images, the sizes of these nanoparticles were estimated, ranging from 5 to 20 nm for nanoAg and from 15 to 40 nm for nanoAu. The use of plasma water affected the polydispersity, both for nanoAg and nanoAu, causing the parameter to increase to 1.00. The UV-Vis spectra also confirmed the observed differences in the polydispersity of the samples. An effect of plasma water on the colour of the produced nanocomposites containing metallic nanoparticles and on the transparency of all samples was also observed. The study showed higher opacity values for non-plasma water-based composites, indicating a lower level of transparency of the films. No significant differences in CO_2_ and H_2_O permeation were observed for the composites produced. An analysis of the FTIR-ATR spectra demonstrated that the type of water used and the synthesis of gold and silver nanoparticles did not lead to changes in the chemical structure of the potato starch. There are no strong interactions between the matrix and the nanoparticles. It can therefore be concluded that starch is a good material for nanoparticle synthesis.

The results obtained indicate that the nanocomposites produced are a promising application material, but further research on the surface and structural properties of starch is needed.

## Figures and Tables

**Table 1:** Nanoparticle size and Polydispersity Index (PDI) in the obtained composites.

Sample	Z-Average (nm)	PDI	Peak 1	Peak 2
PS/Ag	1431.00	0.91	81.42	18.58
PSP/Ag	536.70	1.00	58.10	23.12
PS/Au	924.00	0.84	78.30	15.59
PSP/Au	1125.00	1.00	66.40	20.62

**Table 2:** Water properties of nanocomposites.

Sample	PS	PSP	PS/Ag	PSP/Ag	PS/Au	PSP/Au
Water content (WC) [%]	13.02 ± 0.30 ^abc^	15.09 ± 0.45 ^bc^	12.17 ± 0.60 ^ac^	13.26 ± 0.70 ^abc^	11.46 ± 3.19 ^ac^	14.12 ± 0.76 ^bc^
Water solubility (WS) [%]	27.42 ± 1.92 ^ab^	27.57 ± 0.53 ^ab^	26.22 ± 0.56 ^a^	27.66 ± 0.86 ^ab^	28.34 ± 1.25 ^ab^	26.55 ± 0.32 ^ab^

The same superscript letters in each column demonstrate a lack of significant difference between values (*p* < 0.05). Values are expressed as mean ± SD.

**Table 3:** Mechanical properties of films.

Sample	Thickness [mm]	TS (MPa)	EAB (%)
PS	0.106 ± 0.018 ^c^	4.50 ± 0.39 ^a^	43.94 ± 1.90 ^a^
PSP	0.094 ± 0.01 ^d^	3.39 ± 0.31 ^b^	26.06 ± 2.25 ^c^
PS/Ag	0.119 ± 0.009 ^ab^	3.80 ± 0.48 ^b^	33.52 ± 4.30 ^b^
PSP/Ag	0.113 ± 0.003 ^bc^	4.44 ± 0.33 ^a^	32.67 ± 3.62 ^b^
PS/Au	0.124 ± 0.003 ^a^	3.48 ± 0.34 ^b^	25.96 ± 3.25 ^c^
PSP/Au	0.123 ± 0.006 ^a^	1.74 ± 0.77 ^c^	6.30 ± 2.91 ^d^

TS—Tensile strength, E—Elongation at break. Values are expressed as mean ± SD. Parameters in columns denoted with the same letters (a, b, etc.) do not differ statistically at the level of confidence 0.05.

**Table 4:** Colour properties of the nanocomposites.

Sample	L*	a*	b*	Opacity
PS	98.50 ± 0.08 ^a^	0.01 ± 0.01 ^d^	2.57 ± 0.04 ^c^	5.33
PSP	98.39 ± 0.05 ^a^	0.02 ± 0.00 ^d^	2.52 ± 0.05 ^c^	4.74
PS/Ag	73.54 ± 0.60 ^b^	13.84 ± 0.54 ^b^	74.63 ± 0.53 ^a^	7.02
PSP/Ag	68.09 ± 0.26 ^c^	24.82 ± 0.37 ^a^	69.30 ± 0.32 ^b^	5.49
PS/Au	48.28 ± 0.38 ^d^	13.81 ± 0.14 ^b^	−16.01 ± 0.04 ^e^	8.14
PSP/Au	46.15 ± 0.22 ^e^	12.56 ± 0.05 ^c^	−13.77 ± 0.07 ^d^	10.39

* Values are expressed as mean ± SD. Parameters in columns denoted with the same letters (a, b, etc.) do not differ statistically at the level of confidence 0.05.

## Data Availability

The data presented in this study are available upon request from the corresponding author.

## Supplementary Materials

The following supporting information can be downloaded at: https://www.mdpi.com/article/10.3390/ijms24032200/s1.
