# Supplementary material for: Comparison of Physicochemical Properties of Silver and Gold Nanocomposites Based on Potato Starch in Distilled and Cold Plasma-Treated Water
Source: Int J Mol Sci. 2023 Jan 22;24(3):2200. doi: 10.3390/ijms24032200 (PMC9916708; doi:10.3390/ijms24032200)
Supplement: Supplementary file 1 [file ijms-24-02200-s001.zip › ijms-2126061-supplementary.pdf]

Comparison of physicochemical properties of silver and gold nanocomposites based on potato starch in distilled and cold plasma-treated water

Magdalena Janik[1], Karen Khachatryan[1*], Gohar Khachatryan[1], Magdalena Krystyjan[1], Zdzisław Oszczęda[2]

[1]Faculty of Food Technology, University of Agriculture, ul. Balicka 122, 30-149 Kraków, Poland;

[2]Nantes Nanotechnological Systems, Dolnych Młynów Street 24, 59-700 Bolesławiec, Poland

*Corresponding author: karen.khachatryan@urk.edu.pl

Supplementary data:

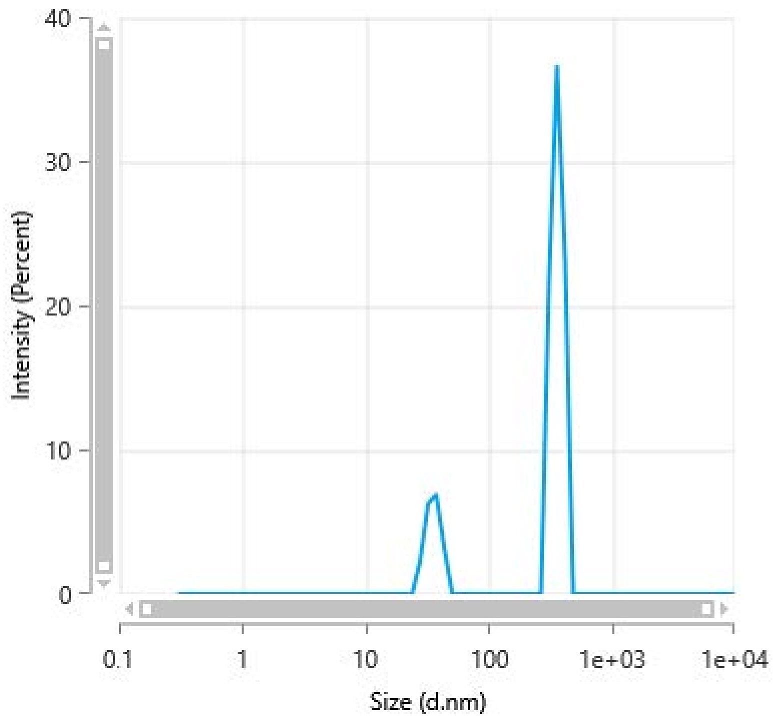

Figure S1. *Size distribution by intensity of PS/Ag.*

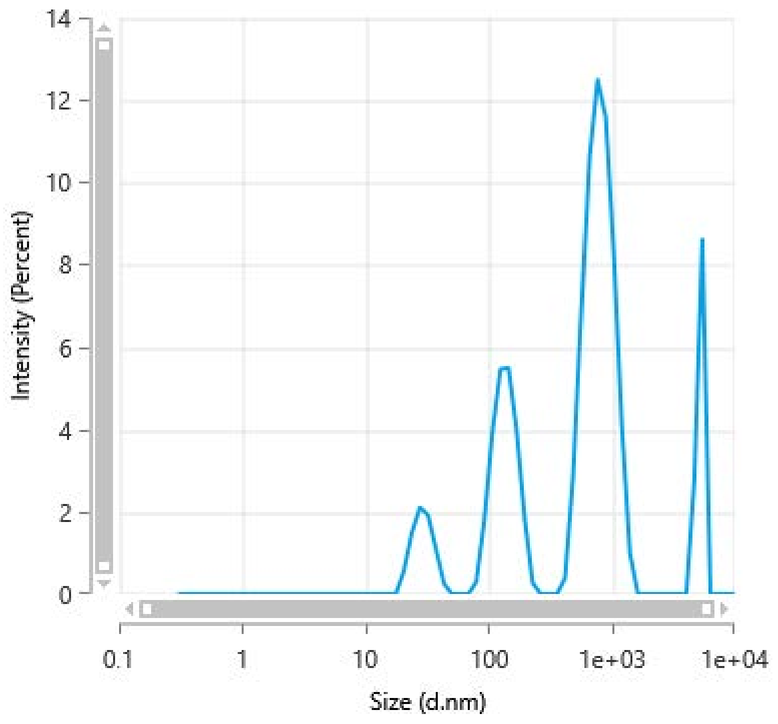

Figure S2. *Size distribution by intensity of PSP/Ag*

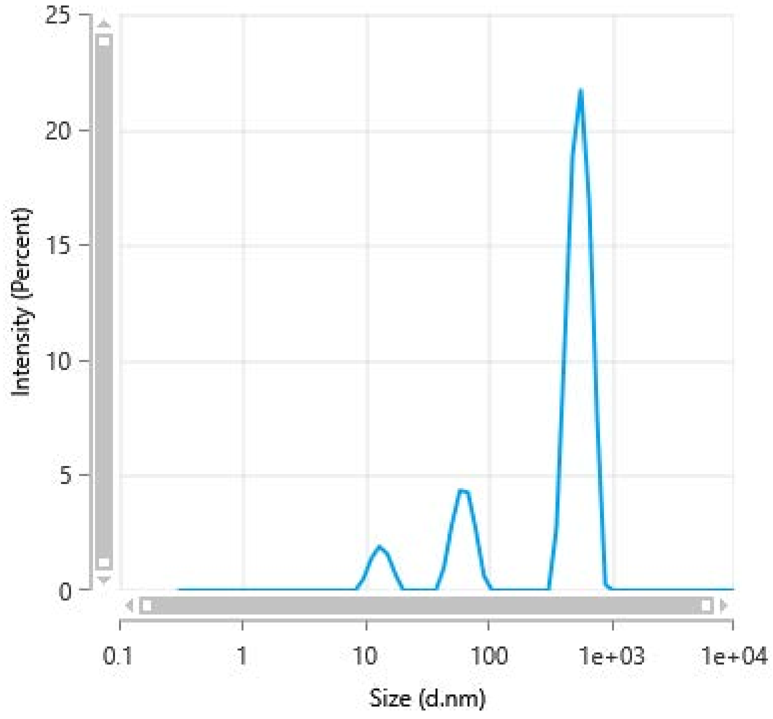

Figure S3. *Size distribution by intensity of PS/Au*

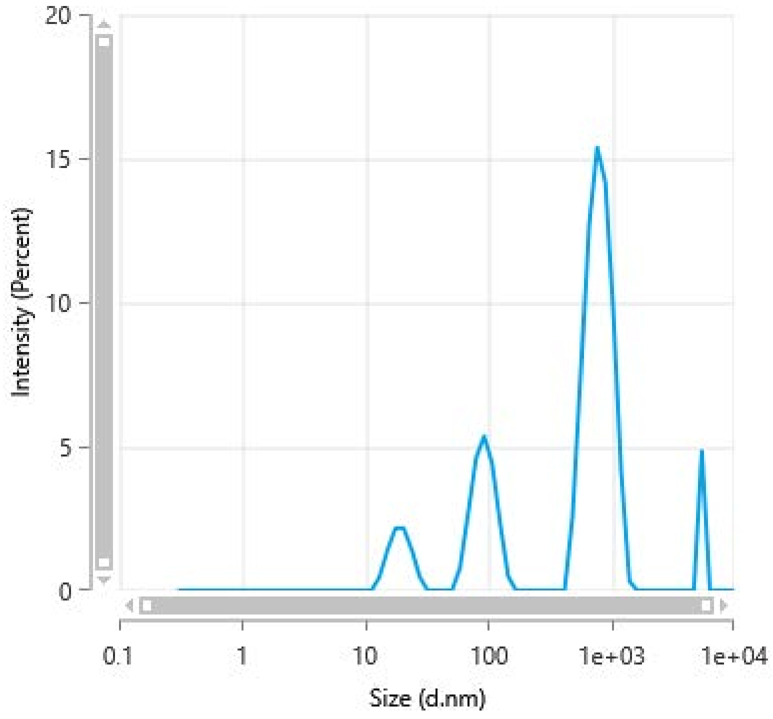

Figure S4. *Size distribution by intensity of PSP/Au*
